# The effect of brewery spent grain application on biogas yields and kinetics in co-digestion with sewage sludge

**DOI:** 10.7717/peerj.10590

**Published:** 2020-12-22

**Authors:** Aleksandra Szaja, Agnieszka Montusiewicz, Magdalena Lebiocka, Marta Bis

**Affiliations:** Faculty of Environmental Engineering, Lublin University of Technology, Lublin, Poland

**Keywords:** Anaerobic co-digestion, Kinetics, Brewery waste, Sewage sludge, Biogas production

## Abstract

The present study examines the effect of introducing dried brewery spent grain (BSG), known as the main solid by-product of the brewery industry on biogas yields and kinetics in co-digestion with sewage sludge (SS). The experiment was conducted in semi-continuous anaerobic reactors (supplied once a day) operating under mesophilic conditions (35°C) at different hydraulic retention times (HRT) of 18 and 20 d. In co-digestion runs, the BSG mass to the feed volume ratio was constant and maintained 1:10.The results indicated that the addition of BSG did not influence the biogas production, by comparison with SS mono-digestion (control run). At HRT of 18 d, in the co-digestion run, the average methane yield was 0.27 m^3^ kg/VS_added_, while in the control run the higher value of 0.29 m^3^ kg/VS_added_was observed. However, there was no difference in terms of statistical significance. At HRT of 20 d, the methane yield was 0.21 m^3^ kg/VS_added_ for both mono- and co-digestion runs. In the BSG presence, the decrease in kinetic constant values was observed. As compared to SS mono-digestion, reductions by 21 and 35% were found at HRT of 20 and 18 d, respectively. However, due to the supplementation of the feedstock with BSG rich in organic compounds, the significantly enhanced energy profits were achieved with the highest value of approx. 40% and related to the longer HRT of 20 d. Importantly, the mono- and co-digestion process proceeded in stable manner. Therefore, the anaerobic co-digestion of SS and BSG might be considered as a cost-effective solution that could contribute to the energy self-efficiency of wastewater treatment plants (WWTPs) and sustainable waste management for breweries.

## Introduction

Currently, one of the main concerns of municipal WWTPs is the high energy consumption and related operational costs. At large WWTPs, SS is commonly stabilized using anaerobic digestion. The biogas generated therein is considered as a renewable energy source that could contribute to the energy self-sufficiency of WWTPs (Panepinto et al., 2016). The enhancement of biogas production may be achieved by introducing an additional substrate into the digesters, and thus managing their unutilized biogas potential. This strategy is known as the anaerobic co-digestion process (AcoD) (Braun, 2002; Mata-Alvarez et al., 2014; Siddique & Wahid, 2018). Apart from the economic issue, the AcoD presents several advantages over mono-digestion (Esposito et al., 2012; Chow et al., 2020). The implementation of an additional substrate could enhance the process stability by improving the nutrient balance and diluting the inhibitory substances in the feedstock (Xie et al., 2016; Hagos et al., 2017). Nevertheless, the synergetic effect of this strategy on microorganisms involved in anaerobic treatment, demands the application of suitable substrates with complementary compositions (Mata-Alvarez et al., 2014; Yang et al., 2019). It is well known that SS is characterized by low organic load, unfavorable C/N ratio and low alkalinity. It also contains AD inhibitors such as heavy metals and xenobiotics. Therefore, SS should be co-digested with substrates with a significant content of easily biodegradable organic matter, high C/N ratio and significant alkalinity. Additionally, the application of products rich in micro- and macro-elements is recommended. Apart from the co-substrate composition, a crucial factor may be the availability on the local market and possible pre-treatment cost (Mata-Alvarez et al., 2014). Thus far, in co-digestion with SS, various organic substrates has been applied e.g., fats, oils and greases (FOG), organic fraction of municipal solid wastes (OFMSW), fruit and vegetable wastes (FWW), agro-industrial by-products namely cheese whey (CW), glycerol (GLY), sugar beet pulp (SBP), distillery waste (DW) and slaughterhouse wastes (SHW). As is shown in Table 1, the implementation of different waste may cause additional operational problems for WWTPs.

**Table 1:** Characteristic of co-substrates used in co-digestion with SS.

**Co-substrate**	**Methane yield** **m**^**3**^**CH**_**4**_**/kg VS**	**C/N ratio**	**Advantages**	**Disadvantages/operational problems**	**References**
FOG	0.7–1.1	10–42	- significant methane potential - beneficial C/N ratio	- high concentration of LCFA (possible inhibition of the methanogenic activity) - clogging in the liquid or gas systems - foaming in digester	Davidsson et al. (2008), Silvestre et al. (2011), Long et al. (2012) and Mata-Alvarez et al. (2014)
OFMSW	0.2–0.7	11–21	- availability at local market - significant content of easily biodegradable organic matter - high C/N ratio	-presence of heavy metal (possible AD process inhibition) - low alkalinity value (lack of buffer capacity) - low content of micro and macro elements - possible pre-treatment	Chow et al. (2020), Campuzano & González-Martínez (2016) and Mata-Alvarez et al. (2014)
Manures	0.15–0.30	6–25	- high alkalinity (ensuring buffer capacity)	-low organic load - high N concentrations (possible inhibition of methanogenesis) - relatively low methane yield	Rabii et al. (2019), Mata-Alvarez et al. (2014) and Li et al. (2018)
FWW	0.15–0.69	25–53	- presence of vitamins, minerals, enzymes - high concentration of easily biodegradable organic matter - beneficial C/N ratio	- seasonal availability - possible pre-treatment to maintain the substrate properties - essential oils content (possible AD inhibition)	Bouallagui, Cheikh & Hamdi (2003), Khan et al. (2015) and Martínez et al. (2018)
SHW	0.7–0.1	<10	- significant methane potential - high content of organic matter - high lipid content	- unfavorable C/N ratio - high concentration of N and LCFA (inhibitors of the methanogenic activity)	Borowski & Kubacki (2015)
GLY	0.35–0.49	78–3000	- significant biodegradability (approx. 100%) - high alkalinity - extreme high C/N ratio and pH (pH 10.3-13) - low N content - small dose (1%)	- possible fast overloading of digester - strict control of AD process	(Aguilar-Aguilar et al. (2017), Hutnan et al. (2013) and Silvestre, Fernández & Bonmatí (2015)
CW	0.28–0.7	acid CW 11–24 sweet CW 2	- significant content of easily biodegradable organic matter (lactose) - presence of vitamins - high protein content - significant methane potential	- sweet CW - low C/N ratio leading to ammonia inhibition - acid CW - possible process inhibition caused by low pH (pH 3.0-4.5)	Kavacik & Topaloglu (2010), Chatzipaschali & Stamatis (2012) and Szaja & Montusiewicz (2019)
DW	0.3–0.7	20–24	- significant content of easily biodegradable organic matter - presence of micro–and macro-nutrients - presence of exogenous amino acids and B vitamins - significant methane potential	- possible process inhibition caused by low pH (pH 3.0-4.5)	Mohana, Acharya & Madamwar (2009), Acharya et al. (2010), Prakash, Sockan & Raju (2014) and Sankaran et al. (2014)
SBP	0.34–0.54	35–40	- beneficial C/N ratio - high content of organic matter - significant methane potential	- presence of hardly biodegradable lignin - possible pre-treatment	Fang, Boe & Angelidaki (2011), Ziemiński & Kowalska-Wentel (2017) and Borowski et al. (2016)
BSG	0.27–0.39	>12	- high content of organic matter - presence of mineral salts, B vitamins and amino acids - beneficial C/N ratio - high buffering capacity	- presence of hardly biodegradable lignin - possible pre-treatment - possible inhibition caused by phenolic compounds	Cater et al. (2005), Dos Santos-Mathias, Moretzsohn de Mello & Camporerese-Sérvulo (2014); Poerschmann et al. (2014), Panjičko et al. (2017), Bougrier et al. (2018) and Szaja & Montusiewicz (2019)

From this group, one of the organic wastes that can be potentially used in anaerobic digestion with SS is brewery spent grain (BSG), the main solid by-product of the brewery industry, generated in large quantities (Mussatto, Dragone & Roberto, 2006). Its global yield is estimated at 38.6 × 10^6^ tons per year and the production accounts for 85% of all waste generated by breweries (Mussatto, 2014; Tan et al., 2019). It is commonly supplied to local farms where it is used as animal feed (Lynch, Steffen & Arendt, 2016) resulting in a small profit for the companies. In some countries, BSG is also disposed of landfills (Buffington, 2014). Importantly, its potential might be used in biotechnological processes (Sturm et al., 2012). According to the UE Waste Framework (EUR-Lex, 2020), such a substrate should be specifically considered as a potential energy source (Ravindran & Jaiswal, 2016). However, the mono-digestion of the BSG is ineffective because of limited biodegradability of lignin which results in low biogas yields and requires extended HRTs (Sežun et al., 2011). The improvement of BSG decomposition may be achieved involving various pre-treatment methods; however, this brings additional financial costs and may lead to the formation of inhibitory intermediates, such as phenolic compounds (Sežun et al., 2011; Kainthola et al., 2019). The afored-mentioned problems might be overcome in the co-digestion process. Thus far, this substrate has been successfully co-digested with Jerusalem artichoke phytomass (Malakhova et al., 2015), cattle dung (Tewelde et al., 2012), cow dung and pig manure (Poulsen, Adelard & Wells, 2017) as well as monoazo dye and glucose/sodium acetate (Gonçalves et al., 2015). Due to its composition and significant biogas potential, it may also be co-digested with SS. Among the different co-substrates (Table 1), the presence of deficient vitamins, mineral salts and amino acids, in particular, may enrich the SS composition, resulting in higher biogas production. Other advantageous features of BSG are its beneficial C/N ratio and high buffering capacity, that may contribute to the stable process performance. Moreover, the application of BSG in anaerobic co-digestion with SS may bring many benefits to both breweries and WWTPs. The implementation of this technology allows for a sustainable and effective management of this waste (Sturm et al., 2012). It should be noted that energy consumption and waste disposal have become a serious problem of many breweries. At the same time, the biogas generated in the AcoD process may be considered as an alternative fuel to generate heat or electricity, ensuring the energy self-sufficiency of WWTP (Wu et al., 2018). Furthermore, the application of BSG does not contribute to the deterioration of digestate composition, which does not exclude its use of agricultural purposes (Mussatto, 2014).

Importantly, the recent study relating to the batch-mode co-digestion of BSG and SS has shown that this process could be effective, improving the biogas potential by 19% (Lebiocka, Montusiewicz & Bis, 2018). Although AcoD brings numerous benefits to WWTPs, it constitutes complex transformations carried out by a consortium of various microorganisms, characterized by different physiology, nutritional needs, growth kinetics and sensitivity to environmental conditions (Chen, Cheng & Creamer, 2008; Lübken et al., 2007; Angelidaki et al., 2011). The AcoD efficiency depends on many factors such as temperature range, pH, C/N ratio, substrate composition, presence of inhibitors as well as operational parameters (Angelidaki et al., 2011; Mao et al., 2015). Unappropriated choice of these can result in some operational problems and may sometimes lead to the process breakdown. Most of the difficulties are caused by inadequate substrate ratios,organic loading rate (OLR) and hydraulic retention time (HRT) (Chow et al., 2020). To avoid any unfavorable effects in existing facilities, the laboratory experiments and the kinetic studies should be performed beforehand. Moreover, co-substrate addition should be strictly controlled by the operators of WWTPs (Gavala, Angelidaki & Ahring, 2003; Manchala et al., 2017).

In laboratory conditions the AD/AcoD may be performed both in batch and semi-continuous mode (Sivakumar, Bhagiyalakshmi & Anbarasu, 2012; Zhang, Su & Tan, 2013). Batch systems are widely used in preliminary studies to evaluate the biomethane potential of different substrates and to conduct toxicity tests (Angelidaki, Ellegaard & Ahring, 1999; Hernandez & Kleinheinz, 2016).

These allow for testing the specific properties of various substrates in a short duration. As compared to the semi-continuous mode, the batch one incurs lower financial costs in relation to the construction of devices (Kothari et al., 2014). However, the results obtained in batch experiments may differ significantly from those achieved in full scale systems (Pilarski et al., 2020). This happens because the effect of fluctuations in the feed composition, HRT and OLR is omitted (Doble & Kumar, 2005). On the other hand, semi-continuous systems are more expensive and time-consuming, but the influence of operational conditions on process efficiency is included when these systems are used. Moreover, these are technologically similar to the digesters used within full-scale WWTPs (Weiland, 2008; Budde et al., 2016). For this reason, the tests conducted in semi-continuous mode provide a greater opportunity for the successful technical implementation of AcoD (Sarker et al., 2019).

The mathematical kinetic models are widely used for predicting and simulating AD performance under various conditions. The application of this tool leads to reduction of the treatment costs, as well as an improvement in process efficiency. It also allows for a quick response to instability of the process, so doing preventing process failure (Angelidaki, Ellegaard & Ahring, 1999; Gavala, Angelidaki & Ahring, 2003; Biswas, Chowdhury & Bhattacharya, 2007; Zhang et al., 2019). The present study examines the effect of introducing the dried BSG on the biogas yields and kinetics in co-digestion with SS using semi-continuous anaerobic digesters. Thus far, such a co-digestion system has not been investigated. Moreover, an energy balance was given to show the potential energy profits generated as a result of the implementation of BSG in WWTPs digesters. The energy aspect of AcoD may be a crucial factor in the decision to apply this strategy to full-scale systems (Carlsson, Lagerkvist & Morgan-Sagastume, 2016; Ruffino et al., 2020).

## Materials & Methods

### Material characteristics

The main substrate (SS) was obtained from the separately thickened sludge from primary and secondary clarifiers at the Puławy WWTP (Poland). The research was conducted on the basis of a contract between the Municipal Water and Sewage Company S.A. in Puławy (WODOCIĄGI PUŁAWSKIE, Skowieszyńska 51, 24-100 Puławy, Poland) and Faculty of Environmental Engineering (Lublin University of Technology). Under laboratory conditions, these were mixed at the recommended volume ratio of 60:40 (primary:wastesludge), homogenized, then screened through a three mm sieve and stored at 4 °C in a laboratory refrigerator for no longer than one week. Before supplying the SS to the reactors, this was kept at ambient temperature indoors until it reached 20 °C (Szaja & Montusiewicz, 2019).

**Figure 1:**
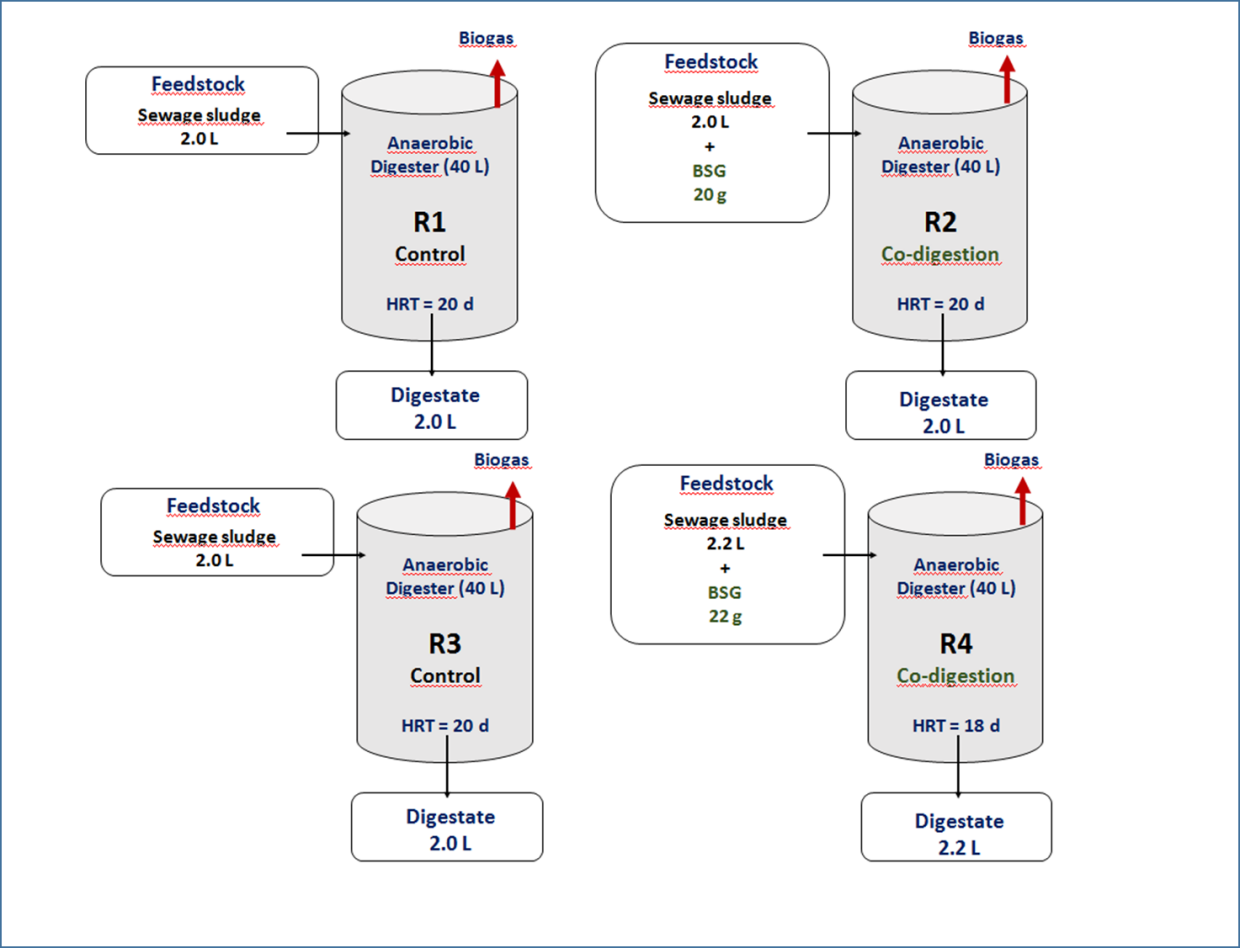
Scheme of operational set-up in experiment.

The BSG was used as a co-substrate to SS. It was obtained from a local brewery, Grodzka 15 in Lublin (Poland). In order to ensure a stable substrate composition, the BSG was dried at 60 °C for 2 h in a laboratory dryer. Then, this sample was milled to a particulate size of 2.0 mm, partitioned in accordance with the assumed doses (Fig. 1) and stored in dry closed boxes. In co-digestion runs, the SS and BSG were homogenized using a low-speed mixer (Szaja & Montusiewicz, 2019). The characteristics of the substrates are presented in Table 2.

**Table 2:** Composition of the substrates used in the experiment.

**Parameter**	**Unit**	**SS**		**BSG**
		Avg. value	Upp./low.95% mean	Avg. value ± SD^a^
COD	mg/L	44227	40286/48168	72623 ± 3144
SCOD	mg/L	2539	1685/3393	–
VFA	mg/L	1143	694/1591	2095 ± 189
pH		6.19	5.93/6.45	6.19 ± 0.64
Alkalinity	mg/L	843	753/933	2967 ± 139
TS	g/kg	37.8	35.2/40.4	223.9 ± 4.3
VS	g/kg	28.3	26.4/30.2	215.1 ± 2.9
TN	mg/L	3942	3431/4452	877 ± 359
TP	mg/L	1115	945/1285	171 ± 97
NH_**4**_^+^-N	mg/L	54.9	36.6/73.3	22.1 ± 6.1
PO_**4**_^3−^-P	mg/L	292.1	55.11/529.18	25.1 ± 6.9

**Notes.**

a SD, standard deviation.

### Experimental set-up—installation and operational set-up

The study was performed in semi-continuous reactors (supplied once a day) operating under mesophilic conditions (35 °C) at different HRTs. The R1 and R3 digesters were supplied with SS only (the control runs), while the R2 and R4 reactors were fed using a mixture of SS and BSG (the AcoD runs). In the AcoD runs, the BSG mass to the feed volume ratio was constant and was maintained at 1:10, but the HRT differed from 20 to 18 d. The detailed operational set-up is presented in Fig. 1 and Table 3.

**Table 3:** The OLR values in experiment.

**Run**	**Feed composition**	**OLR**	
		**Avg**	**Upp./low.95% mean**
		kg VS/m^3^d	
R1	SS (control)	1.35	1.23/1.46
R2	SS + BSG	1.73	1.68/1.78
R3	SS (control)	1.49	1.41/1.58
R4	SS + BSG	1.98	1.84/2.13

**Figure 2:**
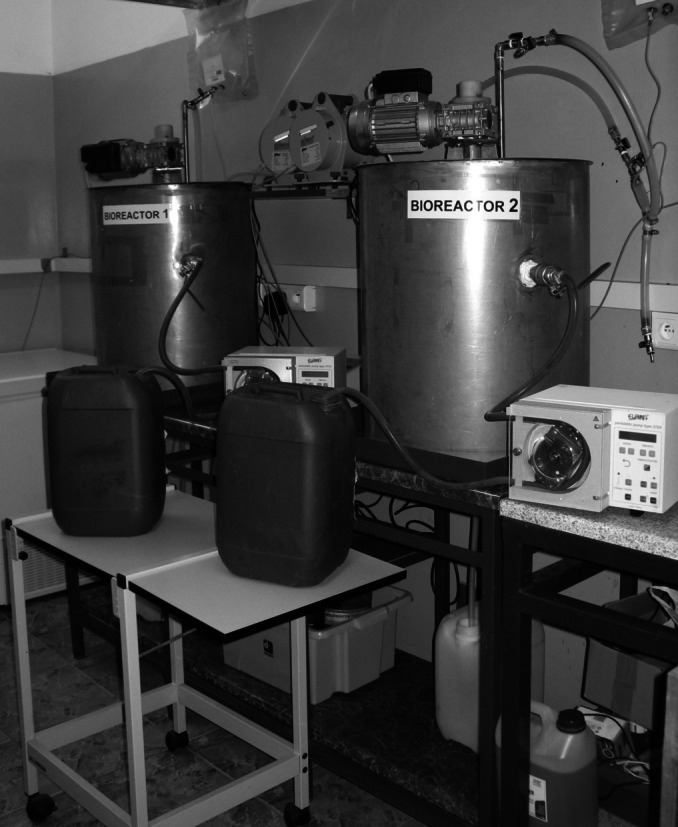
Laboratory installation used in experiment.

The laboratory installation is shown in Fig. 2. Each reactor had an active volume of 40 L, while the volume of head space was 20 L. To maintain a stable temperature each digester was equipped with a heating jacket. The gas installation consisted of a digital mass flow meter, gaseous pipes, a gas sampler, pressure equalization tank and valves. The feedstock was provided to the reactor using a peristaltic pump. Moreover, each reactor had storage vessels both for the feedstock and digestate. Mixing was carried out using a mechanical stirrer with a rotational speed of 50 1/min (Szaja & Montusiewicz, 2019).

An inoculum for all reactors was collected from the mesophilic anaerobic digester with a volume of 2500 m^3^, operating at HRT of 25 d at the Puławy WWTP. The equalized sample of such a digestate was immediately transported to the laboratory, then divided into four parts supplying, each reactor with a volume of 40 L. Throughout the adaptation phase that lasted 30 d, all reactors were operated without feeding to ensure inoculum post-digestion, indicated by slight daily biogas production (0.01NL/d). As a result, the following inoculum characteristic was achieved: TS of 19.7 ± 0.26 g/kg, VS of 12.2 ± 0.44 g/kg and pH of 8.37 ± 0.01. After adaptation, the experiments of semi-continuous AD/Aco-D started. Every day the digesters were supplied by the feedstock according to the adopted schedule (Fig. 1), with an analogous volume of digestate discharged from them. The semi-continuous experiment lasted 90 for days, including 30 days for microorganism acclimatization to the specific feedstock composition and 60 days for the measurements.

### Analytical methods

The analytical methods used in the present study were as previously described in Szaja & Montusiewicz (2019). The SS composition was controlled once a week, while the BSG characteristic was determined once for the whole experiment. The analyses were carried out immediately after the substrate delivery. The following parameters were monitored for both the SS and BSG: total chemical oxygen demand (COD), total solids (TS), volatile solids (VS), total nitrogen (TN), total phosphorus (TP), VFA, alkalinity, pH level, ammonia nitrogen (NH_4_^+^ −N) and orthophosphate phosphorus (PO_4_^3−^ −P). These were performed with Hach Lange UV–VIS DR 5000 (Hach, Loveland, CO, USA) according to Hach analytical methods (hach.com). Additionally, soluble chemical oxygen demand (SCOD) was determined for the SS using the aforementioned method. The pH values were controlled by the HQ 40D Hach-Lange multimeter (Hach, Loveland, CO, USA). Total and volatile solids were performed in accordance with the Standard Methods for the Examination of Water and Wastewater (APHA, 2005).

The feedstock composition was controlled once a week, whereas the digestate was analyzed twice a week. For both, the analogous parameters were determined: COD, TS, VS, VFA, alkalinity, and pH.

Biogas production was estimated every day using an Aalborg (Orangeburg, NY, USA) digital mass flow meter. Its composition (CH_4_, CO_2_, N_2_ and H_2_S) was measured using a ThermoTrace GC-Ultra (Thermo Fisher Scientific, Milan, Italy) gas chromatograph coupled with a conductivity detector fitted with divinylbenzene (DVB) packed columns (RTQ-Bond). The assay procedure was consistent with themanual of this device (assets.thermofisher.com)

The parameters applied in the analysis were 50 °C for the injector and 100 °C for the detector. The carrier gas was helium with a flux rate of 1.5 cm^3^/min(restek.com). The peak areas were determined by means of the computer integration program (CHROM-CARD).

The kinetics of biogas production were evaluated by determining the constant of the biogas production rateand the untapped biogas potential. The latter parameter represents the difference between the maximum biogas production that can theoretically be obtained from a portion of feedstock introduced to the digester every day(V_max_) and the related experimental value achieved from the system for continuous data acquisition (V_e_). The biogas production curves were constructed on the basis of the averaged experimental data acquired from an XFM Control Terminal. The biogas production was described using a first-order kinetic equation (Gavala, Angelidaki & Ahring, 2003):

(1) \begin{eqnarray*}{V}_{f}={V}_{max} \left[ 1-exp \left( -k\cdot t \right) \right] \end{eqnarray*}

where V_f_ is the biogas volume in time (L), k is a constant of the biogas production rate (1/h) and t is the operational time (h). This method is typically applied for kinetics evaluation in batch systems. However, it was also successfully adopted for semi-continuous reactors (Szaja & Montusiewicz, 2019). In the present study, high values of the determination coefficients (R^2^) were achieved confirming the accuracy of such an approach.

### Statistical analysis

The biogas production curves required for evaluating kinetics were prepared based on the averaged experimental data downloaded from an XFM Control Terminal. The kinetic parameters, such as the constant of the biogas production rate and the maximum biogas production were calculated involving a nonlinear regression method. The strength of the relationships between the results achieved experimentally and those obtained using the equation of the first-order reaction, were established using Pearson’s correlation coefficient (R) and determination coefficient (R^2^). The statistical analysis was conducted by ANOVA (Shapiro–Wilk’s, Levene’s and Tukey’s tests were included) with StatsoftStatistica software (v 13). The differences were assumed to be statistically significant at *p* < 0.05.

## Results and Discussion

### Removal efficiency of organic compounds

**Figure 3:**
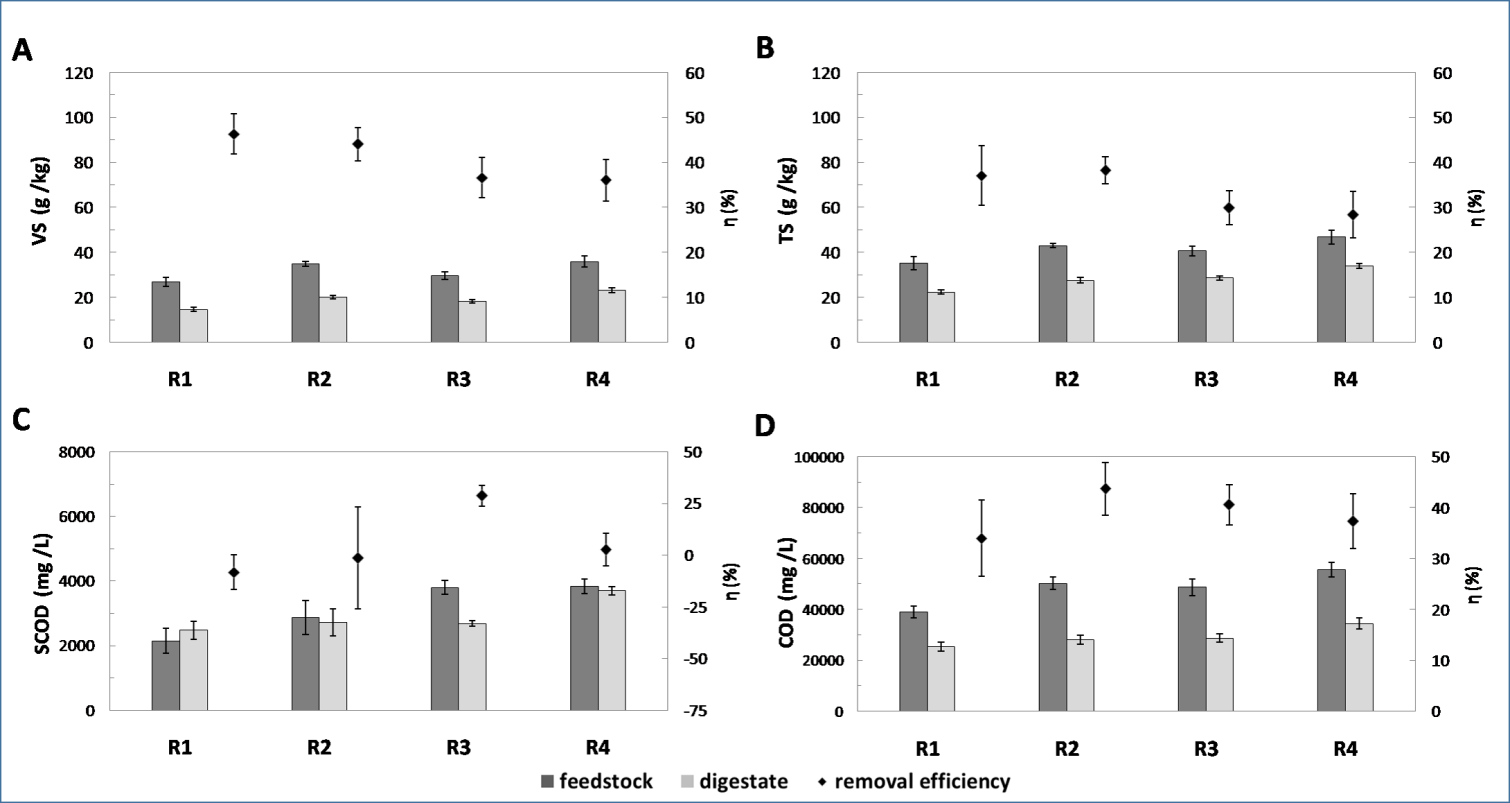
Concentration of VS (A), TS (B), SCOD (C) and COD (D), in feedstock and digestate, as well as related removal efficiencies (average values are reported, error bars represent 95% confidence limits for means).

The application of BSG improved the feedstock composition as compared to SS (Fig. 3) and the differences were of statistical significance for VS, TS and COD. In comparison to the control runs, the VS content was enhanced by 29.6 and 21.1% in R2 and R4, respectively, with the related average values of 34.8 and 35.9 g/kg. For SS this was only 26.9 and 29.7 g/kg (in R1 and R3, respectively).

A similar tendency was observed for TS concentration. In this case, the TS improvements were 22.4 and 15.4%, while the TS concentration reached 43 and 46.9 g/kg for R2 and R4, respectively. In the control runs the TS content was much lower and constituted 35.1 and 29.7 g/kg for R1 and R3, respectively.

Considering the COD, the average values in co-digestion runs were 50.4 (R2) and 55.7 (R4) g/L. The SS was characterized by a significantly lower concentration of 39.2 (R1) and 48.8 (R3) g/L. In relation to the control runs, the increases of 28.6 and 14.2% were found in R2 and R4, respectively. Analogously, in the BSG presence the soluble fraction of chemical oxygen demand (SCOD) in the feedstock was enhanced (Fig. 3C), however, the observed differences were of no statistical significance. In this case, the SCOD reached 2,880 and 3,842 mg/L in R2 and R4, respectively, while for the SS the average values were 2147 and 3,800 mg/L (in R1 and R3, respectively).

Moreover, the application of BSG also significantly increased the OLR, and the major difference was observed at shortened HRT of 18 d. Therein, the enhancement reached 33% as compared to the control, while at HRT of 20 d the related improvement was 28% (Table 3). The observed improvements in the feedstock characteristic were due to the BSG composition (Table 2). As compared to SS, such a co-substrate had a significantly higher content of organic matter. This fact should contribute to enhancing process efficiency in the co-digestion systems.

Despite the improved feedstock composition in the presence of BSG, VS removal decreased to 44.1 and 36.1% at HRT of 20 and 18 d, respectively, whereas in the controls the average values were 46.3 (R1) and 36.6% (R3) (Fig. 3A). Interestingly, a major decline was noted when HRT were shortened to 18 d. The observed tendency might indicate the process inhibition caused by significant VFA concentration in the BSG (Table 2). Its high concentration in AD may lead to a decrease in pH value resulting in acidification and the creation of conditions which are especially toxic for methanogens (Murto, Bjórnsson & Mattiasson, 2004), thus contributing to a decrease in methane production and finally to the process failure (Angelidaki, Ellegaard & Ahring, 1999; Chen, Cheng & Creamer, 2008; Franke-Whittle et al., 2014). Moreover, the possible occurrence of phenolic compounds, formed through BSG drying and milling may affect the process performance (Sežun et al., 2011; Retfalvi, Tukacs-Hajos & Szabo, 2013; Sawatdeenarunat et al., 2015). It is known that phenolic compounds may damage microbial cells by affecting the membrane permeability, resulting in leakage of intracellular components and the deactivation of the enzymatic systems (Monlau et al., 2014; Milledge, Nielsen & Harvey, 2019).

Regarding the TS removal, the slight increase from 37.1 (R1) to 38.2% (R2) was found at HRT of 20 d. Conversely, shortening HRT to 18 d led to a minor decline from 29.9 (R3) to 28.4% (R4). Though, it should be pointed out that the differences noted were of no statistical significance. In co-digestion run at HRT of 20 d, the improvement in both SCOD and COD removal occurred as compared to SS mono-digestion (Figs. 3C, 3D). At HRT of 18 d a diminishing tendency was found, and a statistically significant decrease was observed for SCOD, which dropped from 28.8 (R3) to 2.7% (R4), respectively. The minor decline from 40.6 (R3) to 37.4% (R4) was found with the COD removal.

Considering the removal efficiencies, shortening the HRT from 20 to 18 d cannot be recommended for the co-digestion of SS with BSG. Generally, the lignocellulosic biomass requires prolonged retention times in comparison to other substrates (Yadvika et al., 2004). This observation might be attributed to the presence of highly resistant and recalcitrant compounds, mainly lignin (Montusiewicz et al., 2017). The BSG complex structure indicated that such a substrate is not easily accessible to AD microbes which are especially associated with hydrolytic bacteria (Khanal, 2008; Sawatdeenarunat et al., 2015). An analogous trait was typical for other lignocellulosic wastes, such as wheat straw (Shi et al., 2017) and maize (Banks, 2004).

### Process stability

The process stability was evaluated by estimating the pH value, alkalinity, VFA concentration and the VFA/alkalinity ratio (Table 4). Considering the feedstock composition, the major differences in the BSG presence were noted for alkalinity and VFA concentration. As compared to the control run (R3), a statistically significant decrease in alkalinity of almost 8% was found only in R4. Conversely, the VFA content increased in the presence of the BSG. The enhancements of 42 and 4.5%occurred in R2 and R4, respectively (Table 4). This tendency was attributed to the implementation of BSG characterized by a large VFA content (Table 2), but the observed differences were of no statistical significance. After anaerobic digestion, a growth in digestate pH was observed. For all runs, the average values were at the levels favorable for methanogens (Table 4). Additionally, the alkalinity increased more than four-fold and the digestate revealed a relatively low VFA concentration, which indicated a stable process performance. However, at shortened HRT of 18 d, a statistically significant increase of digestate VFA content was found, as compared to the control run. This effect might result from a possible digester overload (Chen, Cheng & Creamer, 2008). The co-digestion stability was confirmed by the values of VFA/alkalinity ratio which increased slightly in the presence of the co-substrate. For both controls, the related average values were 0.12, while in co-digestion runs these reached 0.13 and 0.16 in R2 and R4, respectively. The results might suggest a minor inhibitory effect of BSG accompanying the HRT shortage (R4). It should be mentioned that for the lignocellulosic biomass, the shortened HRT might also contribute to the instability of the process (Shi et al., 2017). Importantly, the VFA/alkalinity ratio still remained lower than 0.3, confirming stable process conditions (Bernard et al., 2001).

**Table 4:** The pH value, alkalinity and VFA concentrations in feedstock and digestate in experiment.

**Run**	**pH**		**Alkalinity mg CaCO**_**3**_**/L**		**VFA mg/L**	
	Feedstock	Digestate	Feedstock	Digestate	Feedstock	Digestate
R1	6.63 6.59/6.68^a^	7.46 7.39/7.52	735 638/832	3218 3178/3258	492 382/603	399 374/424
R2	6.6 6.51/6.69	7.76 7.62/7.91	785 700/870	3293 3131/3454	700 554/845	425 382/468
R3	5.84 5.74/5.93	7.38 7.22/7.54	931 898/966	3828 3802/3853	1698 1350/2045	445 430/461
R4	5.66 5.57/5.76	7.32 7.19/7.44	859 825/894	3525 3484/3566	1780 1649/1912	571 550/592

**Notes.**

a data represent lower/upper 95% means.

### Biogas production and its kinetics

The supplementation of SS with BSG did not influence the biogas production (Table 5). Due to the BSG characteristics, including a significant amount of carbohydrates, a decreased methane content was observed and the major divergence was found at HRT of 18 d. Importantly, despite the reduction observed, biogas with such a characteristic may still be efficiently used at WWTPs in combined heat and power units (CHP). Consequently, in this case a diminished methane yield was observed and the average values were 0.29 and 0.27 m^3^CH_4_/kgVS_added_ in control and co-digestion runs, respectively, but the difference was of no statistical significance. At HRT of 20 d, the methane yield was 0.21 m^3^/kgVS_added_ for both reactors. These results exceeded the values reported in different studies. Zou et al. (2018) investigated the anaerobic co-digestion of residual sludge and various lignocellulosic wastes in batch mode; therein, the specific methane yields varied between 0.13–0.16 m^3^CH_4_/kg VS_added_. However, these yields were significantly enhanced as compared to the sewage sludge mono-digestion. Comparing other wastes co-digested with SS, different values of biogas/methane production were found. In the co-digestion of SS and slaughterhouse waste, the highest biomethane production reached 0.55m^3^CH_4_/kgVS_added_ (Salehiyoun et al., 2020). By using SS and an organic fraction of municipal solid wastes, biogas production varied between 0.4–0.6 m^3^/kgVS_added_ (Sosnowski, Wieczorek & Ledakowicz, 2003).

**Table 5:** Biogas yields as well as methane content in experiments (average value and 95% confidence limits are given).

**Parameter**	**Unit**	**R1**	**R2**	**R3**	**R4**
Biogas yield	m^3^/kgVS_added_	0.39 ± 0.05	0.40 ± 0.04	0.5 ± 0.05	0.5 ± 0.05
	m^3^/kgTS_added_	0.30 ± 0.03	0.33 ± 0.03	0.36 ± 0.03	0.38 ± 0.03
	m^3^/kgVS_removed_	0.88 ± 0.16	0.96 ± 0,2	1.46 ± 0.30	1.53 ± 0.49
	m^3^/kgTS_removed_	0.90 ± 0.25	0.88 ± 0,14	1.30 ± 0.26	1.64 ± 0.68
	m^3^/kgCOD_removed_	0.60 ± 0.14	0.56 ± 0.07	0.78 ± 0.12	0.90 ± 0.16
Methane content	%	54.12 ± 0.64	52.16 ± 0.60	56.99 ± 0.49	54.75 ± 0.45

**Figure 4:**
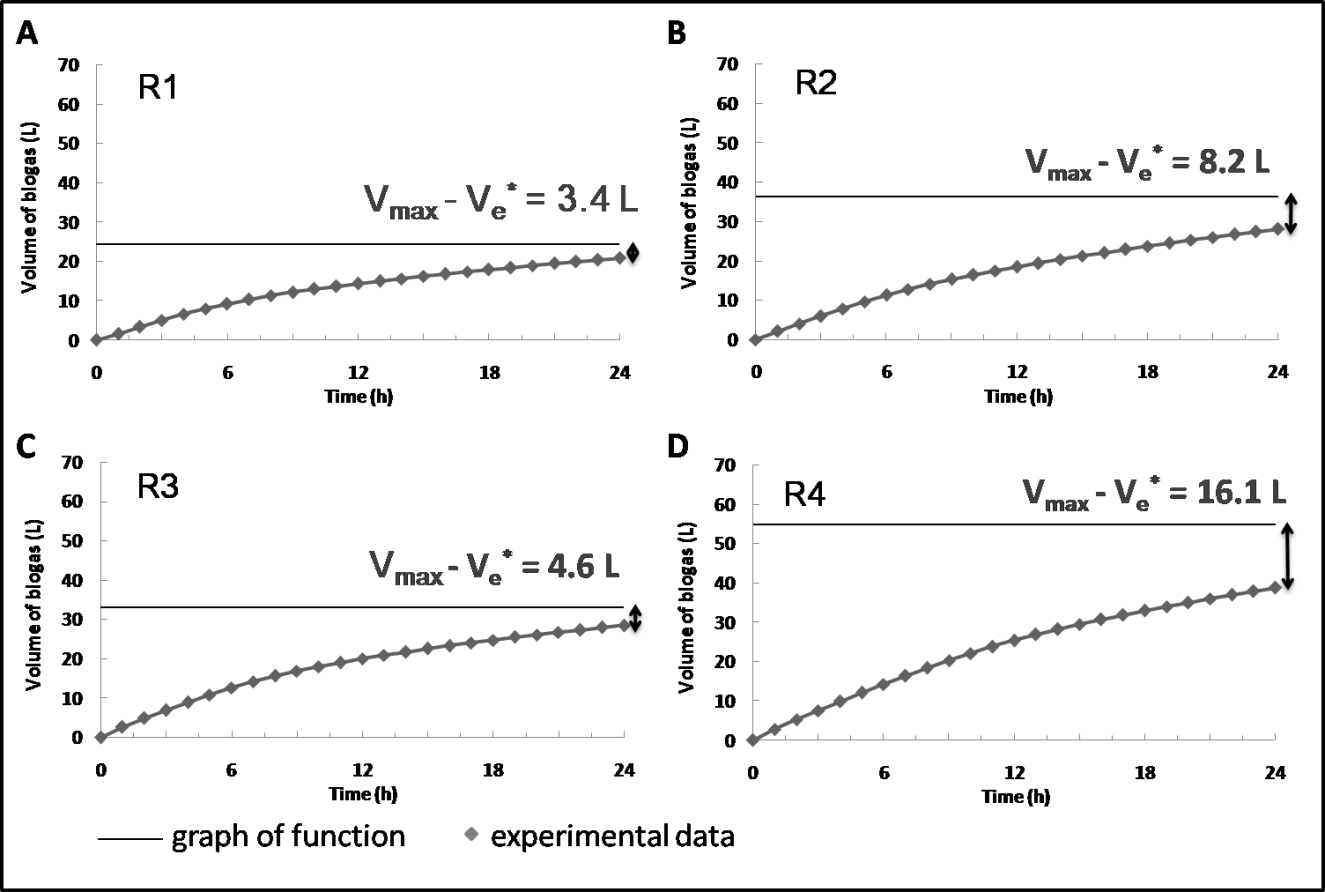
Biogas production in time in R1 (A) R2 (B) R3 (C) and R4 (D) (the average values from 30 measurement days are reported).

Considering kinetics, in the present study, the semi-continuous system was applied. The reactor was supplied once a day with the portion of substrate or substrates and simultaneously the same volume of digested medium was removed from it. Accordingly, the biogas production between each feeding related to a temporal interval 0–24 h (Fig. 4) (Szaja & Montusiewicz, 2019).

The supplementation of the feedstock with BSG led to a decrease in the constant of biogas production rates and a simultaneous increase of untapped biogas potential (Table 6). In comparison to SS mono-digestion, the k values dropped by 21 and 35% at HRT of 20 and 18 d, respectively. Simultaneously, the related untapped biogas potential was 2.5 and 3.5 times greater compared to the control, which indicated that using lignocellulosic matter as a co-substrate needed ensuring the prolonged, rather than shortened HRT. Li et al. (2013) noted that feedstocks with significant lignin content (more than 15% on TS basis) were characterized by low first-order rate constant as compared to other lignocellulosic and manure wastes. This tendency might be attributed to the presence of recalcitrant compounds and the inhibitory effects of phenolic compounds potentially appearing during the BSG pretreatment (milling and drying) (Retfalvi, Tukacs-Hajos & Szabo, 2013; Panjičko et al., 2017). It should also be noticed that the hydrolysis of lignocellulose constitutes the rate-limiting step through the conventional AD process (Khanal, 2008).

**Table 6:** The average values of kinetics constants as well as coefficients of determination.

**Parameter**	**Units**	**R1**	**R2**	**R3**	**R4**
Daily biogas production	NL/d	21.26 ± 3.64	28.13 ± 3.15	28.69 ± 2.60	38.41 ± 2.39
Constant of biogas production rate k	1/h	0.076	0.060	0.078	0.051
Maximum biogas production V_max_	L	24.32	36.29	33.12	54.83
Untapped biogas potential V_max_-V_e_	L	3.4	8.2	4.6	16.1
Coefficient of determination R^2^	–	0.9993	0.9997	0.9997	0.9999

### Energy balance

The energy balance was estimated on the basis of experimental data for a digester operating at the WWTP in Puławy (Poland). A detailed procedure of its calculation was adopted from the authors’ previous study (Szaja & Montusiewicz, 2019). Interestingly, in the presence of the BSG , significantly enhanced energy profits were found (Table 7). This trend most likely came from the improvement of the feedstock composition through the application of BSG, rich in organic compounds.

**Table 7:** The energy balance calculations of selected runs in experiment.

**Parameter**	**Unit**	**R1**	**R2**	**R3**	**R4**
**Input data**					
VS	g/kg	26.9	34.8	29.7	35.9
Feedstock density	kg/m	1000.5	992.7	1006.4	1002.1
VS load	kg/d	3360	4322	3730	4501
Methane yield	m^3^CH_4_ /kgVS_add_	0.21	0.21	0.29	0.27
Daily methane production	m^3^ CH_4_/d	705.7	914.8	1065.2	1356.2
Feedstock temperature in winter	° C	8	8	8	8
Feedstock flow rate	m^3^/d	125	125	125	139
**Energy balance**					
Theoretical thermal energy	MJ/d	25264	32752	38136	48553
Thermal energy for heating the feedstock	MJ/d	14175	14175	14175	15763
Thermal energy for covering the heat loss	MJ/d	3766	3766	3766	3766
Thermal energy demand	MJ/d	19735	19735	19735	21481
Profit of thermal energy	%	28	66	93.2	126
Net thermal energy profit^a^	%	**37.9**		**32.8**	
Daily energy production	kWh/d	7057	9148	10652	13562
Energy production	kWh/t	56	73	85	98
Theoretical thermal power production	kW	126	164	191	243
Theoretical electric power production	kW	111.7	144.9	168.7	214.7
Profit of theoretic thermal and electric power production	%	**29.6**		**27.4**	

**Notes.**

a Difference of thermal energy demand between the control and co-digestion run.

However, more beneficial results were obtained using longer HRT of 20 d. As compared to the SS mono-digestion, the thermal energy profit was enhanced by approx. 38 and 33% at HRT of 20 and 18 d, respectively, whereas the profit of theoretical thermal and electric power productions was improved by approx. 30 and 27.5% at HRT of 20 and 18 d, respectively. To sum up, the energy generated in the co-digestion system could completely cover the WWTP energy demand, therefore its surplus may be sold to other recipients, increasing company profits in this way.

## Conclusions

The BSG significantly enriched feedstock composition regarding VS, TS and COD. Despite this fact, the application of such a substrate in co-digestion with SS did not affect biogas production efficiency. Comparable biogas yields were found in both mono- and co-digestion runs. However, a negative effect on kinetics was observed in the presence of BSG and a major decline was observed for shortened HRT of 18 d which seems to indicate the need to extend HRT. Importantly, the application of BSG rich in organic compounds significantly enhanced energy profits. Regardless of the HRT, a stable process performance was maintained in co-digestion runs. Therefore, the anaerobic co-digestion of SS and BSG might be considered as a cost-effective solution that could contribute to the energy self-efficiency of WWTPs and sustainable waste management. However, due to the occurrence of hardly degradable compounds (mainly lignin), HRT longer than 18 d is recommended.

##  Supplemental Information

10.7717/peerj.10590/supp-1 Supplemental Information 1 The detailed data in all runs.

## Additional Information and Declarations

### Field Study Permissions

The following information was supplied relating to field study approvals (i.e., approving body and any reference numbers): The sewage sludge was taken form the Puławy municipal wastewater treatment plant (WWTP), Poland. The research was conducted on the basis of a contract between the Puławy municipal wastewater treatment plant and Faculty of Environmental Engineering (Lublin University of Technology). The permission to entry into the WWTP area was issued by the facility Manager, Mr. Kwiatek. While, the sewage sludge was provided by the main Technologist of WWTP Puławy, Mr. Jaśkowski.

### Data Availability

The following information was supplied regarding data availability: Raw data, including detailed data regarding biogas production and its composition, feedstock and digestate analyses, are available as a Supplemental File.
